# Long term effects of fetal undernutrition on rat heart. Role of hypertension and oxidative stress

**DOI:** 10.1371/journal.pone.0171544

**Published:** 2017-02-17

**Authors:** Pilar Rodríguez-Rodríguez, Angel L. López de Pablo, Concha F. García-Prieto, Beatriz Somoza, Begoña Quintana-Villamandos, José J. Gómez de Diego, Perla Y. Gutierrez-Arzapalo, David Ramiro-Cortijo, M. Carmen González, Silvia M. Arribas

**Affiliations:** 1 Departamento de Fisiología, Facultad de Medicina; Universidad Autónoma de Madrid, Madrid, Spain; 2 Departamento de Ciencias Experimentales y de la Salud; Facultad de Farmacia, Universidad CEU-San Pablo, Madrid, Spain; 3 Departamento de Anestesiología y Reanimación; Hospital General Universitario Gregorio Marañón, Madrid, Spain; 4 Departamento de Cardiología, Hospital Clínico San Carlos, Madrid, Spain; Universidade do Estado do Rio de Janeiro, BRAZIL

## Abstract

**Background and aims:**

Fetal undernutrition is a risk factor for heart disease in both genders, despite the protection of women against hypertension development. Using a rat model of maternal undernutrition (MUN) we aimed to assess possible sex differences in the development of cardiac alterations and the implication of hypertension and cardiac oxidative stress.

**Methods:**

Male and female offspring from rats fed *ad libitum* (control) or with 50% of the normal daily intake during the second half of gestation (MUN) were used. Heart weight/body weight ratio (HW/BW), hemodynamic parameters (anaesthetized rats) and plasma brain natriuretic peptide (BNP, ELISA) were assessed in 21-day, 6-month and 22-month old rats. Plasma testosterone (ELISA) and cardiac protein expression of enzymes related to reactive oxygen species synthesis (p22^phox^, xanthine-oxidase) and degradation (catalase, Cu/Zn-SOD, Mn-SOD, Ec-SOD) were evaluated in 21-day and 6-month old rats (Western Blot). Heart structure and function was studied at the age of 22 months (echocardiography).

**Results:**

At the age of 21 days MUN males exhibited significantly larger HW/BW and cardiac p22^phox^ expression while females had reduced p22^phox^ expression, compared to their respective sex-matched controls. At the age of 6-months, MUN males showed significantly larger blood pressure and cardiac xanthine-oxidase expression; MUN females were normotensive and had a lower cardiac expression of antioxidant enzymes, compared to their respective sex-matched controls. At the age of 22 months, both MUN males and females showed larger HW/BW and left ventricular mass and lower ejection fraction compared to sex-matched controls; only MUN males exhibited hypertension and a larger plasma BNP compared to aged male controls.

**Conclusions:**

1) During perinatal life females exposed to fetal undernutrition are protected from cardiac alterations, but in ageing they exhibit ventricular hypertrophy and functional loss, like MUN males; 2) cardiac oxidative stress might be implicated in the observed heart alterations in both sexes and 3) the severity of cardiac damage might be greater in males due to hypertension.

## Introduction

Low birth weight -due to inadequate maternal nutrition, placental alterations or other causes-is associated with several adverse outcomes in adult life; a process known as fetal programming. Among others, low birth weight due to fetal stress has been linked to the development of heart diseases and some of its risk factors such as hypertension [1–5]. Epidemiological studies have been corroborated in experimental animals showing evidence that hypoxia, nutrient deficiency or other stressors during intrauterine life, induce left ventricular hypertrophy (LVH) [6,7], increased susceptibility to ischemia-reperfusion injury [8,9] or arrhythmias [10].

Despite the well known differences between men and women in cardiovascular physiology [11] and the influence of sex in the development and progression of disease, sex as biological variable is not taken into account sufficiently [12]. In the clinical setting this leads to underestimation of the risk of heart disease in women and also to inadequate treatment [13]. Regarding the influence of sex on fetal programming, there is some evidence pointing at a lower impact of fetal programming on hypertension development in women [14]. Despite this fact, cohort studies have evidenced that the association between low birth weight and adult heart disease is comparable in men and women [15,16], particularly after menopause [17]. Therefore, the impact of ageing and the mechanisms implicated in the sex differences in fetal programming of heart disease are not completely elucidated and deserve further studies [18].

Hypertension is one of the possible links between fetal stress and heart disease development. On one hand, hypertension is one of the cardiovascular risk factors most consistently associated with low birth weight [3,14]. Secondly, high blood pressure is a well known stimulus for LVH and myocardial ischemia [8,9]. Furthermore, fetal programming of hypertension exhibits a sexual dimorphism and studies in animal models have demonstrated that females exposed to several stress factors during intrauterine life do not develop hypertension or exhibit milder forms [14,19–21]. This finding has led to exclude females from the majority of studies and few have evaluated the impact of fetal stress on female heart, particularly in ageing.

Oxidative stress is another possible mechanism which might contribute to fetal programming of cardiac disease. The heart is an active source of reactive oxygen species (ROS) since it is the organ with the highest oxygen uptake and largest density of mitochondria and, therefore, it might be particularly vulnerable to redox disbalance. In addition, there is evidence that oxidative stress is implicated in cardiac remodeling [22,23]. Oxidative stress associated to fetal programming is also influence by sex [18]. In this regard, we have evidence, in a rat model of maternal undernutrition during pregnancy (MUN), that females exhibit a better plasma antioxidant status at weaning, while males have a reduced antioxidant capacity and increased plasma carbonyl levels already at the age of 21 days. Furthermore, in this rat model, males, but not females developed hypertension at adult age [24]. Given the role of hypertension and oxidative stress in cardiac structural and functional alterations, in the present study we hypothesize that MUN females might be less susceptible to develop long-term cardiac alterations particularly if they remain normotensive at advanced age. The aims of the present study were to test this hypothesis, assessing in MUN rats at different age points: 1) the development of alterations in cardiac structure and function, through echocardiography and brain natriuretic peptide (BNP) measurements and 2) the role of hypertension and cardiac oxidative stress, through assessment of hemodynamic parameters and heart enzymatic systems responsible for ROS production and degradation. We have also evaluated the possible implication of testosterone in the sex-differences observed in this rat model of fetal programming.

## Materials and methods

Experiments were performed in Sprague Dawley rats from the colony maintained at the Animal House facility of the Universidad Autónoma de Madrid. All experimental procedures conformed to the Guidelines for the Care and Use of Laboratory Animals (NIH publication No. 85–23, revised in 1996), the Spanish legislation (RD 1201/2005) and they were approved by the Ethics Review Board of Universidad Autónoma de Madrid and of the local Government (Comunidad Autónoma de Madrid).

The rats were housed in buckets 36.5/21.5/18.5 cm (length/width/height) on aspen wood bedding and maintained under controlled conditions (temperature 22°C, relative humidity 40% and 12/12 light/dark photoperiod). They were fed with breeding diet (EuroRodent Diet 22; 5LF5, Labdiet, Spain) containing 55% carbohydrates, 22% protein, 4.4% fat, 4.1% fiber and 5.4% mineral, being sodium content 0.26%. Drinking water was provided *ad libitum* in all cases. The animals were free from any pathogen that could interact with the study. The health and welfare of the animals was monitored by staff at least once a day.

### Maternal Undernutrition Model (MUN)

The model of global nutrient restriction in Sprague Dawley pregnant rats (MUN) was established as previously described [24]. Briefly, day 1 of gestation was determined in the dams by observation of sperm in the vaginal smear. Thereafter, they were allocated to one of the experimental groups: control, fed *ad libitum* throughout pregnancy and lactation (C, n = 5 dams) and MUN, fed *ad libitum* during the first 10 days of gestation and with 50% of the averaged control daily intake from day 11 to the end of pregnancy, returning to *ad libitum* diet during lactation (MUN, n = 5 dams). 24h after birth the pups were sexed and weighed individually and the litter was standardized to 12 individuals, 6 males and 6 females if possible (smaller litters were not used).

### Experimental procedures

To avoid litter effect, each experimental protocol described below was performed in male and female offspring from the different litters. Experiments were performed in rats of the following ages: 21 days, 6 months and 22 months.

### Intra-arterial blood pressure measurements

Intra-arterial blood pressure was assessed as previously described [25]. Briefly, the rats were anaesthetized with 37.5 mg/kg Ketamine hydrochloride and 0.25 mg/kg Medetomidine hydrochloride i.p. Thereafter, a cannula was inserted in the iliac artery and the pressure wave was recorded through a pressure transducer (Statham; Harvard Apparatus) on a PowerLab system (ADInstruments) during approximately 60 minutes. Diastolic and systolic blood pressures (DBP, SBP) and heart rate (HR) were measured in the trace, averaging the data from approximately 1 min recording period and mean arterial pressure (MAP) and pulse pressure (PP) were calculated.

### Western blot

Hearts were excised, atria removed, weighted and quickly snap frozen to quantify protein expression of NADPH oxidase subunit p22^phox^, xanthine oxidase (XO), catalase and superoxide dismutase (SOD) isoforms (Cu-Zn-SOD, Mn-SOD and extracellular, EC-SOD). Western blotting was performed as previously described [26]. Briefly, 30 μg protein samples were separated by 15% SDS-PAGE gels. Primary antibodies against p22^phox^ (Santa Cruz Biotechnology; Germany; 1:400 final dilution), Cu/Zn-SOD and Mn-SOD (SantaCruz Biotechnology; Germany; 1:1000 final dilution), EC-SOD (Enzo Life Sciences; USA; 1:1000 final dilution); XO (Santa Cruz Biotechnology; Germany; 1:1000 final dilution) and catalase (Sigma-Aldrich; USA; 1:2000 final dilution) were applied overnight at 4°C. After washing, secondary antibodies (anti-rabbit or anti-mouse IgG-peroxidase conjugated) were applied for 1 h. Blots were washed, incubated in commercial enhanced chemiluminescence reagents (ECL Prime, Amersham Bioscence, UK) and bands were detected by ChemiDoc XRS+Imaging System (Bio-Rad, USA). To prove equal loadings of samples, blots were re-incubated with GADPH antibody (Sigma-Aldrich; USA; 1:3000 final dilution). Blots were quantified using Image Lab 3.0 software (Bio-Rad, USA). Expression values ofp22^phox^, Cu/Zn-SOD, Mn-SOD, EC-SOD, XO and catalase were normalized with GADPH to account for variations in gel loading. Examples of gels used for densitometric analysis are shown in S1 Fig.

### Transtoracic Echocardiography (TTE)

TTE was performed in 22 month-old rats as previously described [27]. Briefly, the rats were anesthetized with 80 mg/kg Ketamine hydrochloride and 10 mg/kg Diazepam, i.p. An Acuson Sequoia C512 system equipped with a 13-MHz transducer was used (Acuson, Mountain View, CA, USA). The images were acquired with the animals in left lateral decubitus. M-mode imaging of the parasternal short-axis (papillary level) view allowed measurement of end-diastolic internal diameter (LVIDd) and end-systolic internal diameter (LVIDs), posterior wall thickness at diastole (PWd) and interventricular septum thickness at diastole (IVSd); HR was also recorded. Values were determined by averaging the measurements of three consecutive cardiac cycles in accordance with the American Society of Echocardiography guidelines. The above measurements were used to calculate left ventricular mass (LVM), expressed in grams, as previously described [28], using the following equation:

$$LVM=0.8\;\left\lfloor 1.04\;{\left(IVSd+LVIDd+PWd\right)}^{3}\text{−}LVIDd^{3}\right\rfloor +0.6$$

### Plasma hormones

*Brain Natriuretic Peptide (BNP)*. Plasma BNP levels were assessed in 21-day, 6-month and 22-month old rats, by a competitive enzyme immunoassay kit (Abbexa rat BNP ELISA kit; UK), according to manufacturer’s instructions. BNP was expressed as pg/ml.

*Testosterone*. Plasma testosterone levels were assessed in 21-day old and 6-month old rats, by a competitive enzyme immunoassay kit (Testosterone ELISA Demeditec Diagnostics GmbH; Germany), according to manufacturer’s instructions. Testosterone was expressed as ng/ml.

### Statistical analysis

Sample size was calculated assuming a probability error of alpha type of 5% (p<0.05) and potency of 80%. Statistical analyses were performed by Graph-Pad Prism (version 5) and SPSS (version 22). The data are expressed as mean ± SEM. Statistical differences between MUN and control rats of each sex were analyzed by Student´s *t* test and the interaction between sex and maternal nutrition was analyzed by 2-way ANOVA.

## Results

### Body weight

24h after birth body weight of MUN rats was significantly smaller compared to controls in both males (MUN = 4.50±0.13g, n = 30; control = 6.48±0.14g, n = 30; p<0.01) and females (MUN = 4.72±0.13g, n = 30; control = 6.73±0.10g, n = 30; p<0.01). During lactation MUN rats accelerated growth and by weaning (21 days old) body weight was not statistically different between MUN and control males (MUN = 49.7±1.4, n = 12; control = 51.3±1.5, n = 12) or between MUN and control females (MUN = 49.1±1.3, n = 12; control = 47.5±1.2, n = 12). At the ages of 6 months and 22 months there was no significant difference between MUN and control male or between MUN and control female rats.

### Heart weight

At the age of 21 days heart weight/body weight ratio (HW/BW) was significantly larger in MUN males compared to control rats, while we did not detect statistical differences between MUN and control females (Table 1). The interaction between sex and fetal nutrition was also statistically significant (Table 2).

**Table 1:** HW/BW at different age points in MUN and control offspring.

	21 days (Weaning)		6 months (Adult)		22 months (Aged)	
	Males	Females	Males	Females	Males	Females
**Control**	4.9±0.09	4.9±0.10	2.7±0.03	2.9±0.07	2.8±0.10	2.7±0.07
	(12)	(12)	(12)	(11)	(7)	(7)
**MUN**	5.5±0.10*	4.8±0.08	2.8±0.05	3.1±0.08	3.6±0.06*	3.0±0.10*
	(12)	(12)	(12)	(11)	(5)	(5)

HW/BW, heart weight/body weight (x1000); Control rats were fed *ad libitum* in pregnancy; MUN rats were exposed to 50% of the normal daily intake during the second half of pregnancy. In parenthesis is shown the number of rats;

*p<0.05 when compared to sex and age-matched control rats.

**Table 2:** Interaction between sex and fetal nutrition for the studied variables.

	21-day old	6-month old	22-month old
Variable	P value	P value	P value
HW/BW	0.013	0.293	0.465
SBP	0.179	0.023	0.066
DBP	0.269	0.098	0.092
MBP	0.429	0.031	0.080
PP	0.969	0.982	0.069
HR (M/K)	0.448	0.604	0.987
HR (D/K)	-	-	0.071
p22phox	0.029	0.889	-
XO	0.689	0.010	-
Cu/Zn-SOD	0.932	0.035	-
Mn-SOD	0.021	0.097	-
EC-SOD	0.184	0.037	-
Catalase	0.412	0.048	-
IVSd	-	-	0.299
PWd	-	-	0.944
LVIDd	-	-	0.378
LVIDs	-	-	0.763
LVM	-	-	0.730
LVEF	-	-	0.583
Testosterone	0.070	0.908	-
BNP	0.666	0.520	0.056

P values for the variables studied, evaluated by 2-way ANOVA. SBP, DBP. MBP, systolic, diastolic and mean blood pressure; PP, pulse pressure; HR (M/K), heart rate under Medetomidine/Ketamine anesthesia; HR (D/K), heart rate under Diazepam/Ketamine anesthesia; HW/BW, heart weight/body weight; XO, xanthine oxidase; SOD, superoxide dismutase; IVSd, interventricular septum at diastole; PWd, posterior wall thickness at diastole; LVIDd, left ventricular internal diameter at diastole; LVIDs, left ventricular internal diameter at systole; LVM, left ventricular mass; LVEF, left ventricular ejection fraction; BNP, brain natriuretic peptide; p< 0.05 was considered statistically significant.

At the age of 6 months no statistical difference in HW/BW were found between MUN and control rats, either in males or females.

At the age of 22 months HW/BW was significantly larger in MUN rats, both in males and in females compared to their respective sex-matched controls (Table 1).

### Haemodynamic parameters

A reduction in blood pressure was observed following Medetomidine/Ketamine anesthesia. After around 30–45 min blood pressure remained stable. This was observed at all ages studied and in all the experimental groups (S2 Fig).

**Fig 1:**
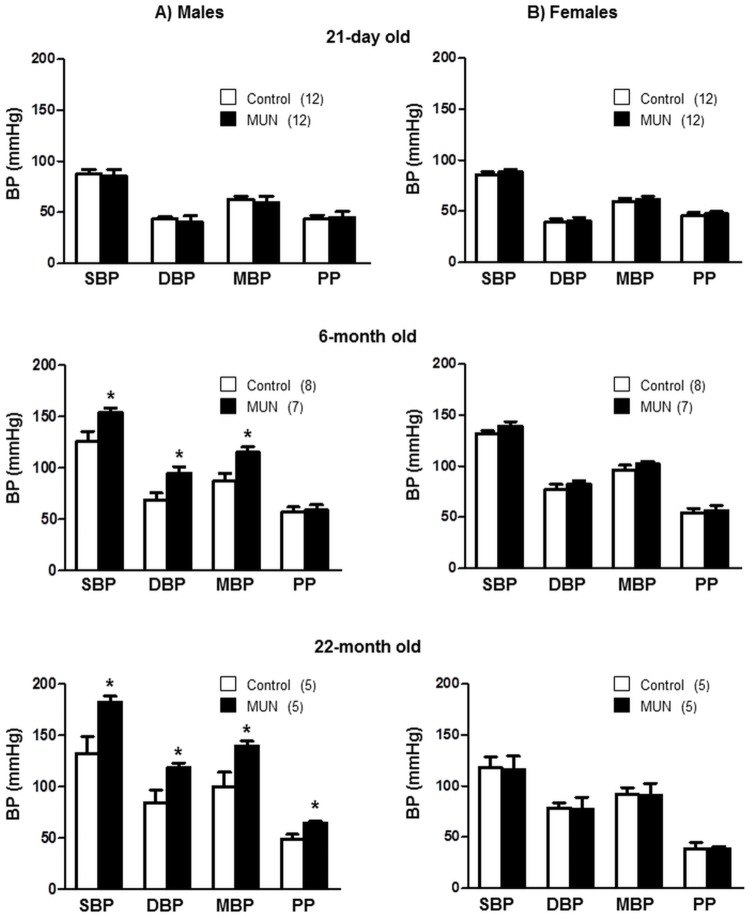
Blood pressure measurements in anesthetized MUN and control rats at different age points. Intra-arterial systolic, diastolic and mean blood pressure (SBP, DBP, MBP) and pulse pressure (PP) in rats under Medetomidine/Ketamine anesthesia. (A) male and (B) female offspring from rats exposed to maternal undernutrition during pregnancy (MUN) and rats fed *ad libitum* (Control). In parenthesis the number or rats used at each age point; *p<0.05 compared to sex and age-matched control rats.

At the age of 21 days we did not detect statistical differences in SBP, DBP, MBP or PP between MUN and control rats, either in males or in females. At the age of 6 months SBP, DBP and MBP, but not PP, were significantly larger in MUN males compared to sex-matched controls (Fig 1A), while we did not detect differences between MUN and control females (Fig 1B). The interaction between sex and maternal nutrition was statistically significant for SBP and MBP. At the age of 22 months MUN males exhibited a significantly larger SBP, DBP, MBP and PP compared to control males (Fig 1A), while we did not detect statistical differences in blood pressure between MUN and control females (Fig 1B). The interaction between sex and fetal nutrition was near statistical significance for SBP and PP (Table 2).

**Fig 2:**
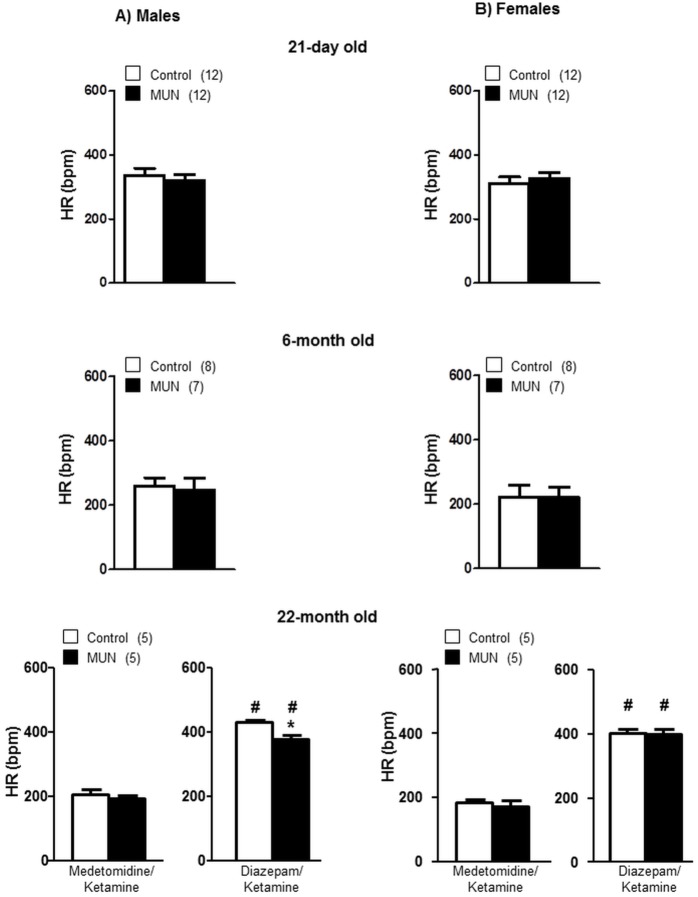
Heart rate (HR) measured in anesthetized MUN and control rats at different age points. HR was measured under Medetomidine/Ketamine anesthesia at all age points. In 22-month old rats HR was also measured under Diazepam/Ketamine anesthesia. (A) male and (B) female offspring from rats exposed to maternal undernutrition during pregnancy (MUN) and rats fed *ad libitum* (Control). In parenthesis the number or rats used at each age point; *p<0.05 compared to sex and age-matched control rats; #p<0.05 compared to Medetomidine/Ketamine anesthesia.

Under Diazepam/Ketamine anesthesia (used for echocardiography study in 22 month old rats) HR was significantly larger compared to HR under Medetomidine/Ketamine. Under Medetomidine/Ketamine anesthesia, HR was not statistically different between MUN and control rats of either sex at any of the ages studied. Under Diacepan/Ketamine anesthesia 22-month old MUN males rats exhibited a significantly reduced HR compared to control males. This difference was not observed in MUN females (Fig 2). The interaction between sex and fetal nutrition was near significance in 22-month old rats (Table 2).

### Expression of enzymes related to ROS production and degradation

**Fig 3:**
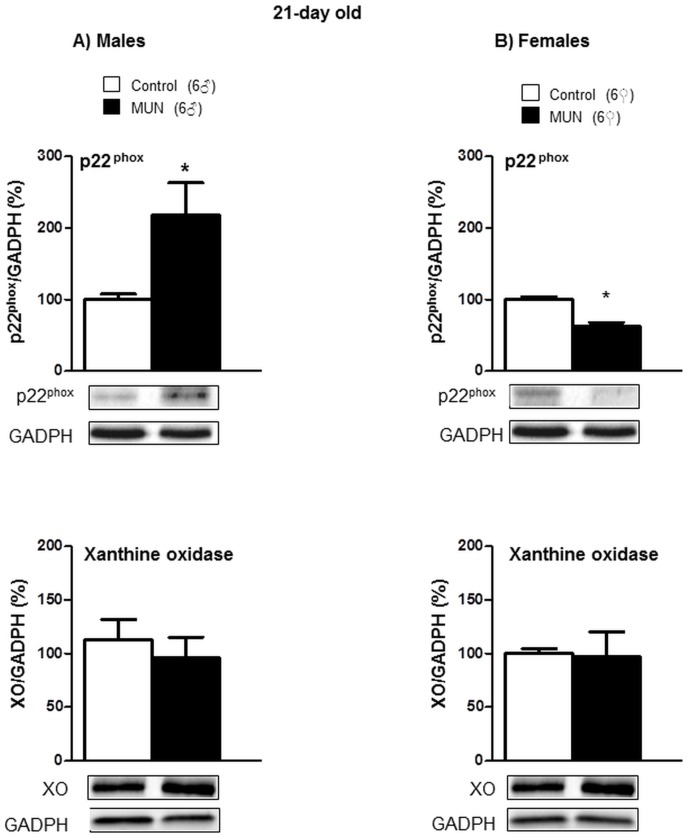
Protein expression of p22^phox^ and xanthine oxidase (XO) in cardiac tissue from 21-day old rats. (A) male and (B) female offspring from rats exposed to maternal undernutrition during pregnancy (MUN) and rats fed *ad libitum* (Control). Diagram bars show the results of densitometric analysis, relativized to GADPH expression. In parenthesis it is shown the number or rats; *p<0.05 compared to sex-matched control rats.

In 21-day old rats, p22^phox^ expression was significantly higher in MUN males compared to sex-matched controls, while we did not detect statistical differences between groups in XO expression (Fig 3A). MUN females exhibited significantly lower expression levels of p22^phox^ compared to female controls; no differences were detected in XO expression between MUN and control females (Fig 3B). The interaction between sex and fetal nutrition was statistically significant for p22^phox^ expression (Table 2).

**Fig 4:**
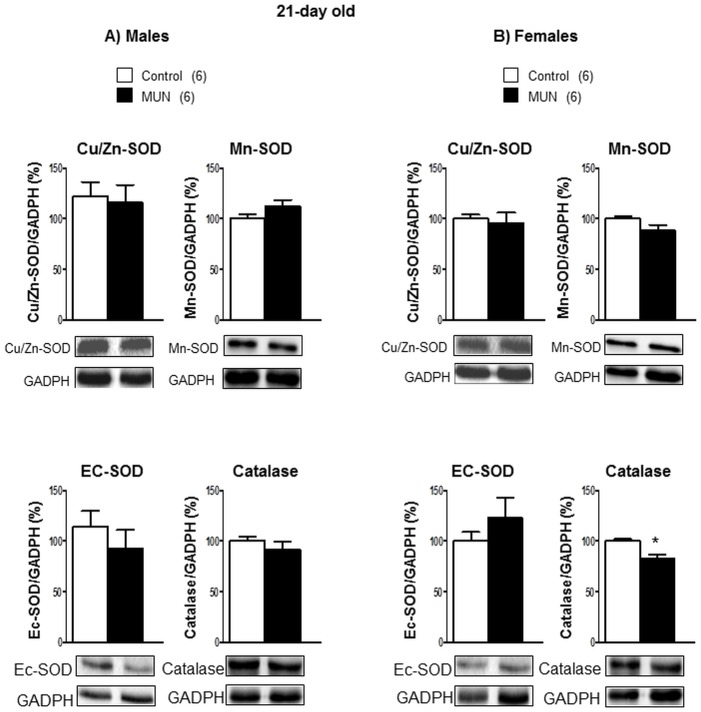
Protein expression of SOD isoforms and catalase in cardiac tissue from 21-day old rats. Superoxide dismutase (SOD) isoforms, Cu/Zn-SOD, Mn-SOD and extracellular SOD (EC-SOD). (A) male and (B) female offspring from rats exposed to maternal undernutrition during pregnancy (MUN) and rats fed *ad libitum* (Control). Diagram bars show the results of densitometric analysis, relativized to GADPH expression. In parenthesis it is shown the number or rats; *p<0.05 compared to sex-matched control rats.

In 21-day old rats we did not detect statistical differences in any of the enzymatic systems responsible for ROS degradation between MUN and control males (Fig 4A). Catalase expression was significantly lower in MUN females compared to sex-matched control rats (Fig 4B). The interaction between sex and fetal nutrition was statistically significant for Mn-SOD expression (Table 2).

**Fig 5:**
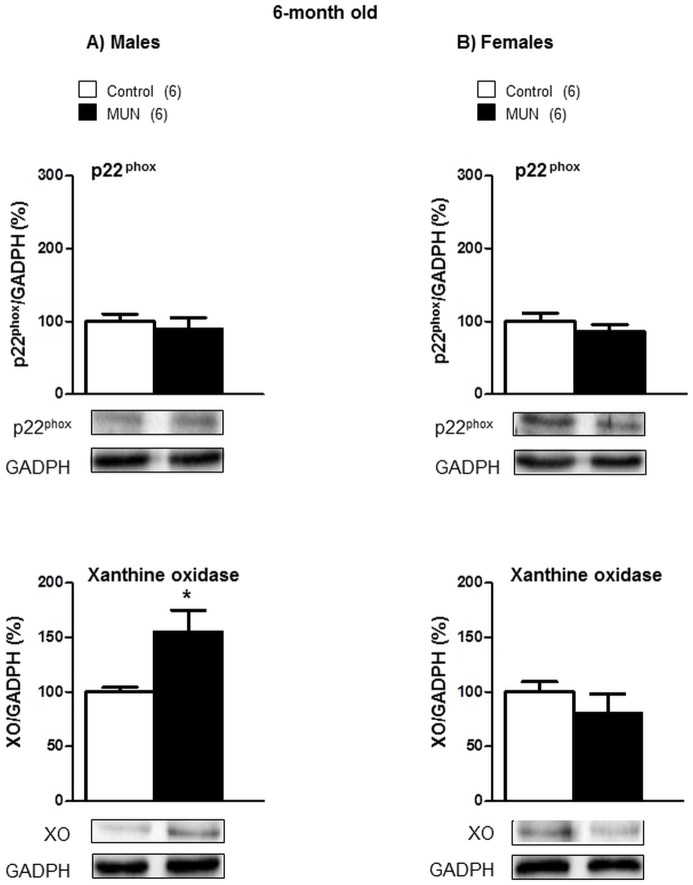
Protein expression of p22^phox^ and xanthine oxidase (XO) in cardiac tissue from 6-month old rats. (A) maleand (B) female offspring from rats exposed to maternal undernutrition during pregnancy (MUN) and rats fed *ad libitum* (Control). Diagram bars show the results of densitometric analysis, relativized to GADPH expression. In parenthesis it is shown the number or rats; *p<0.05 compared to sex-matched control rats.

At the age of 6 months, there was no statistical difference in p22^phox^ expression between MUN and control rats males. However, a significantly higher XO protein expression levels were found in MUN males compared to sex-matched controls (Fig 5A). Regarding females, no statistical differences were detected between MUN and control rats in either p22^phox^ or XO expression (Fig 5B). The interaction between sex and fetal nutrition was statistically significant for XO expression (Table 2).

**Fig 6:**
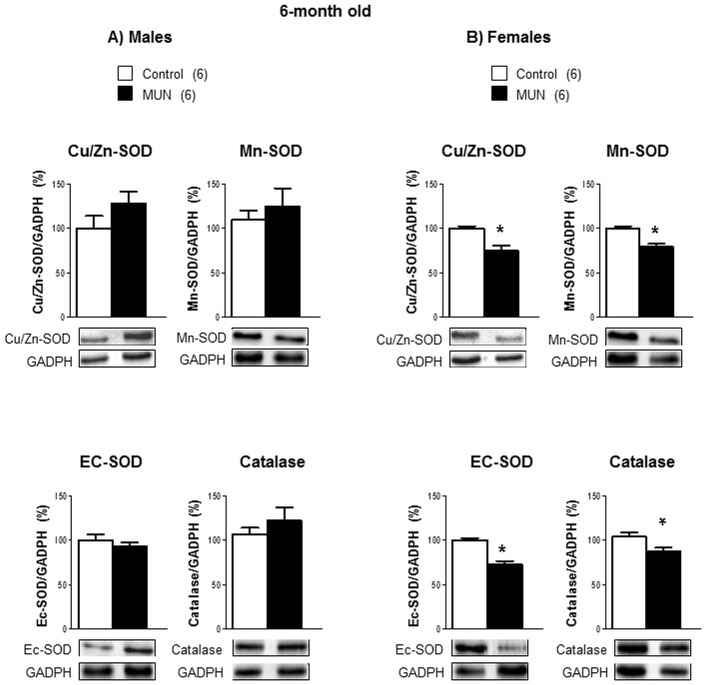
Protein expression of SOD isoforms and catalase in cardiac tissue from 6-month old rats. Superoxide dismutase (SOD) isoforms, Cu/Zn-SOD, Mn-SOD and extracellular SOD (EC-SOD) and catalase. (A) maleand (B) female offspring from rats exposed to maternal undernutrition during pregnancy (MUN) and rats fed *ad libitum* (Control). Diagram bars show the results of densitometric analysis, relativized to GADPH expression. In parenthesis it is shown the number or rats; *p<0.05 compared to sex-matched control rats.

In 6-month old rats, we did not detect statistical differences between MUN and control males in any of the enzymes related to ROS degradation (Fig 6A). Female MUN rats had a significantly lower expression of all the enzymatic systems evaluated compared to female controls (Fig 6B). The interaction between sex and fetal nutrition was statistically significant for Cu/Zn-SOD, EC-SOD and catalase expression (Table 2).

### Transthoracic Echocardiography

**Fig 7:**
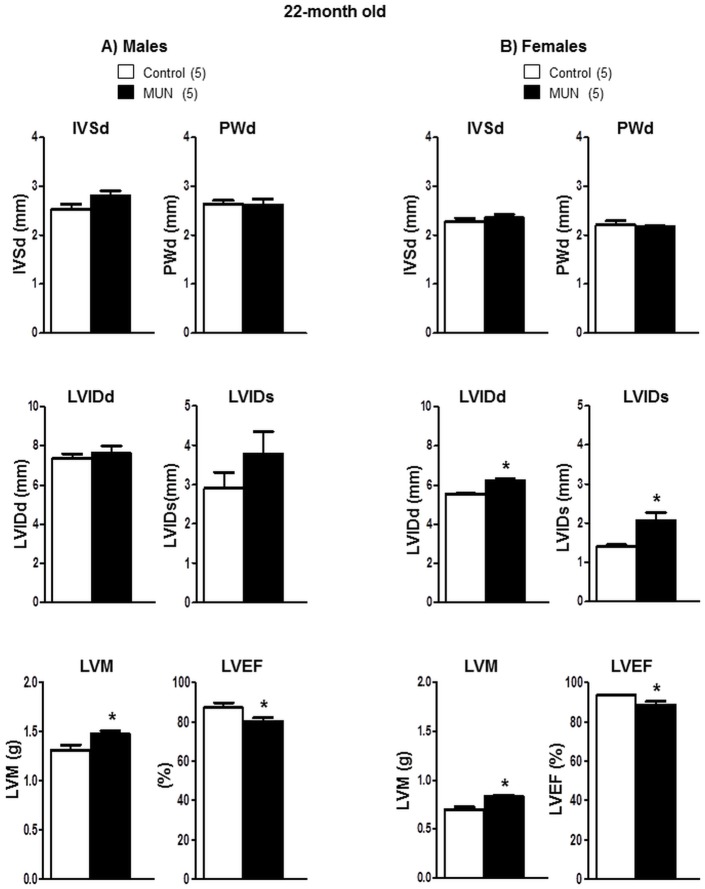
Echocardiographic parameters in 22-month old rats. (A) male and (B) female offspring from rats exposed to maternal undernutrition during pregnancy (MUN) and rats fed *ad libitum* (Control). Interventricular septum at diastole (IVSd), posterior wall thickness at diastole (PWd), left ventricular internal diameter at diastole (LVIDd) and at systole (LVIDs), left ventricular mass (LVM) and left ventricular ejection fraction (LVEF). In parenthesis it is shown the number of rats; *p<0.05 compared to sex-matched control rats.

At the age of 22 months MUN males showed no differences in PWd, IVSd, LVIDd or LVIDs, but a significantly larger LVM compared to controls. MUN males also exhibited a significantly lower ejection fraction compared to sex-matched controls (Fig 7A).

At old age, PWd, IVSd, were not significantly different between MUN and control females. However, LVIDd and LVIDs were significantly larger in MUN females compared to controls. In addition, a significantly larger LVM and smaller ejection fraction was observed in MUN females compared to female controls (Fig 7B).

### Plasma hormones

**Fig 8:**
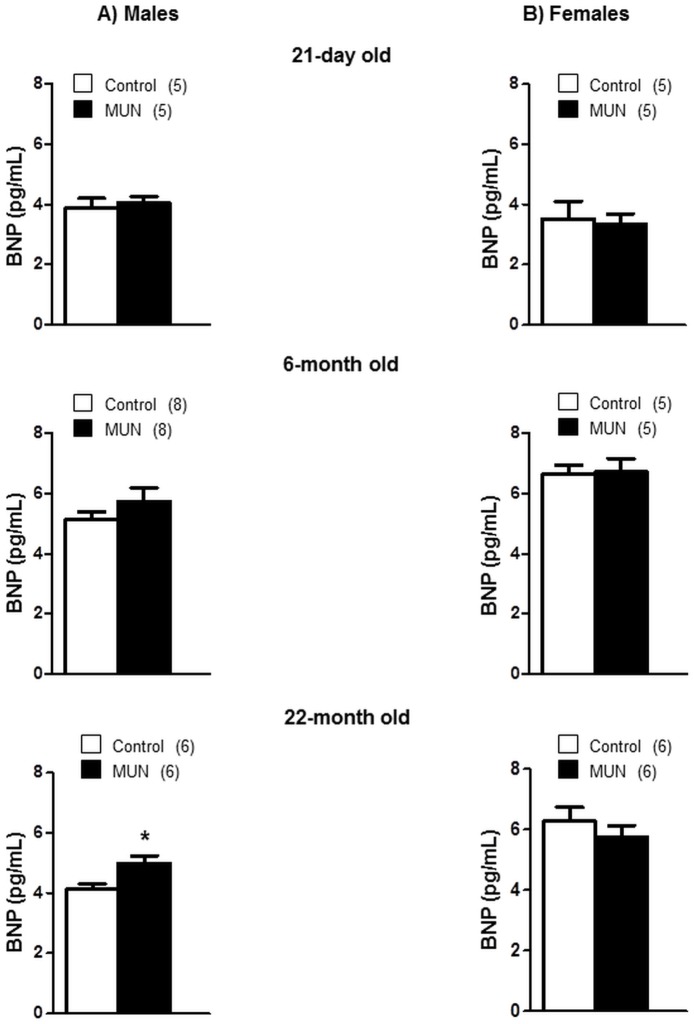
Plasma brain natriuretic peptide (BNP) levels in MUN and control rats of different ages. (A) maleand (B) female offspring from rats exposed to maternal undernutrition during pregnancy (MUN) and rats fed *ad libitum* (Control). In parenthesis it is shown the number of rats; *p<0.05 compared to sex-matched control rats.

In 21-day old rats BNP plasma levels were not significantly different between MUN and control males or females. Similarly, we did not detect statistical differences between MUN and control rats at the age of 6 months either in males or in females. At the age of 22 months, MUN male rats exhibited significantly larger plasma BNP levels compared to male controls. No significant differences were found in females (Fig 8). The interaction between sex and fetal nutrition was near significance at the age of 22 months (p = 0.056, Table 2).

**Fig 9:**
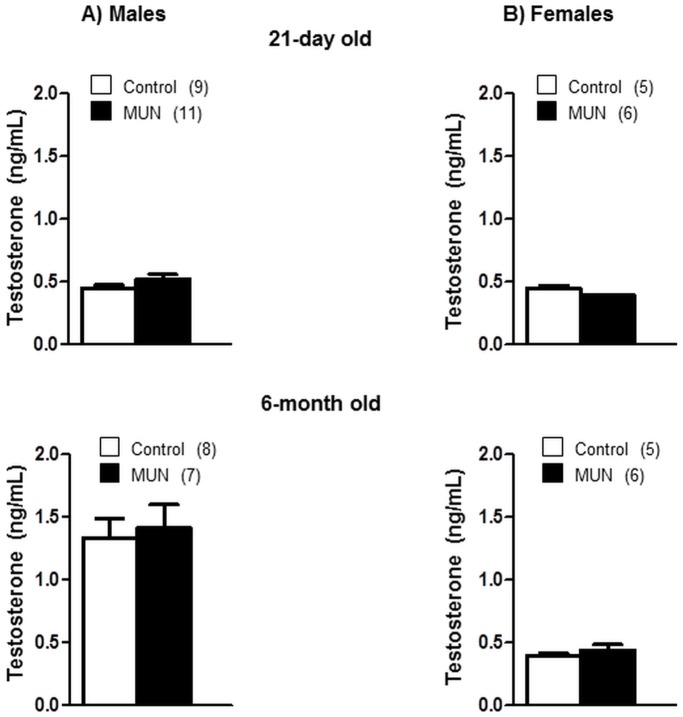
Plasma testosterone levels in 21-day and 6-month old rats. (A) male and (B) female offspring from rats exposed to maternal undernutrition during pregnancy (MUN) and rats fed *ad libitum* (Control). In parenthesis it is shown the number of rats.

At the age of 21 days we did not detect statistical differences in testosterone levels between MUN and control males or between MUN and control females, although there was a tendency towards higher levels in MUN males (Fig 9) and the interaction between sex and fetal nutrition was near statistical significance (p = 0.07, Table 2). At the age of 6 months testosterone levels were larger in male rats compared to 21-day old rats, with no significant difference between MUN and control rats. In 6-month old female rats testosterone levels remained low with no statistical differences between MUN and control rats (Fig 9).

## Discussion

Epidemiological studies worldwide have demonstrated that sub-optimal fetal growth is associated to later development of cardiovascular diseases and some of its risk factors, particularly hypertension. Despite the protection of women against fetal programming of hypertension, the association between low birth weight and heart disease is similar in men and women, particularly when the factor ageing is taken into account [15–17]. Therefore, the influence of sex on fetal programming of heart disease and the role of hypertension as risk factor is not completely elucidated. This is likely due to the difficulty to conduct longitudinal studies in humans and the number of variables which might obscure the interpretation of results. In animal models, which provide a good mean to perform long-term studies controlling environmental influences, there is clear evidence of sex influence in hypertension programming, but few studies have evaluated cardiac alterations taking into account the variable sex and ageing process. Therefore, the present work was conducted to shed light on this aspect, using a rat model of low birth weight induced by maternal global nutrient restriction. Our main findings are that: 1) in male rats fetal undernutrition induced early LVH, sustained hypertension from adult to old age and a reduction in left ventricular function in ageing; 2) females exposed to fetal undernutrition did not develop early LVH, remained normotensive throughout life, but also developed left ventricular functional loss in old age; 3) cardiac tissue oxidative disbalance might contribute to the observed heart alterations in both sexes and 4) cardiac damage at old age might be greater in males due to the presence of sustained hypertension.

MUN males developed LVH in the perinatal period, which is a critical developmental window during which the alterations induced by undernutrition in fetal life might further develop. One of the consequences of fetal undernutrition is a reduction in cell number or functional units in several tissues. In this sense, fetus or newborn animals exposed to fetal stress exhibit reduced cardiomyocyte number [29,30]. Thereafter, during postnatal development in a context of accelerated weight gain, hypertrophic responses and increased HW/BW ratio have been reported in several animal models at weaning [6,7,31,32]. We suggest that a similar process took place in MUN male rats. Despite a similar acceleration of weight in both sexes during the perinatal period, only MUN males exhibited heart hypertrophy. Hypertension can be ruled out as possible stimulus, since at the age of 21 days both MUN males and females were still normotensive. We suggest that this early cardiac hypertrophy might be related to an excess of ROS production by NADPH oxidase—an enzyme implicated in LVH [22]- based on the observed elevation of cardiac p^22phox^ expression in MUN males. Similar findings have been previously demonstrated in male offspring from rats exposed to protein restriction in pregnancy, followed by postnatal accelerated growth, where LVH was associated with cardiac oxidative and nitrosative damage [32]. In the present study we did not evaluate markers of heart oxidative stress. However, we suggest it might also be present, based on our previous finding that MUN males, but not females, had increased plasma carbonyls at weaning [24], a parameter which exhibits a good correlation with protein oxidative damage in cardiac tissue [33].

Regarding the possible mechanism behind an early induction of NADPH oxidase in MUN males, we evaluated the possible role of testosterone, which has been implicated in sex-differences in fetal programming of hypertension [14]. Testosterone has been shown to alter renin-angiotensin-system (RAS) expression in a sex-specific manner [34] and it is well known that angiotensin II activates NADPH oxidase through AT_1_receptors [35]. Although we did not detect statistical differences in plasma testosterone at the age of 21 days, MUN males tended to have higher levels compared to male controls. Thereafter, in adult life, there were no differences in testosterone levels between MUN and control rats. We suggest that testosterone might have been higher in MUN males during the fetal period. To the best of our knowledge, there are no reports on fetal testosterone levels in animal models of fetal undernutrition, but there is evidence that testosterone influences placental and fetal growth with a sex-dependent pattern. For example, maternal testosterone elevation during pregnancy has been shown to induce intrauterine growth restriction [36] and a decrease in uterine arterial blood flow and placental growth, with a greater effect on males [37]. In this regard, we have previous evidence of differences between MUN males and females in placental and fetal growth at the end of gestation [38].

The molecular mechanisms responsible for the predisposition to cardiovascular disease after exposure to fetal stress are not completely elucidated. Among others, epigenetic processes—including alterations in DNA methylation, histone acetylation or non coding microRNAs- have been suggested to play a role. Furthermore, epigenetic modulation has been suggested to be responsible for cardiovascular disease transmission from one generation to another in animals exposed to fetal stress (18). In cardiac tissue, differential expression of miRNA has been reported in males exposed to global nutrient deficiency and suggested to play a role in cardiac hypertrophy (6). On the other hand, the placenta also seems to be sensitive to maternal nutrition in terms of epigenetic marks and a clear sexual dimorphism in the placental response to maternal environment has been reported [39]. The contribution of epigenetic modulation to the sex-differences in fetal programming of CVD deserves further analysis.

MUN males developed hypertension in adult age, as previously demonstrated in other rat models of fetal stress [20] and our previous report [24]. We also found that blood pressure remained elevated until ageing in MUN males, while females remained normotensive at the age of 22 months. Heart rate was not different between MUN and control rats, either in males or females, in young or adult age. However, HR was significantly reduced in 22 month old MUN males compared to controls. We only detected this difference in rats anesthetized with Diazepam/Ketamine (used as anesthesia for Echocardiography) and not in rats under Medetomidine/Ketamine anesthesia. We also found that HR was lower in animals under Medetomidine/Ketamine compared to Diazepam /Ketamine anesthesia. This is likely due to the alpha 2-adrenergic agonist effects of Medetomidine. It has been reported that this anesthesia reduces noradrenaline outflow within the central nervous system, dampening the central sympathetic tone and resulting in bradycardia [40,41]. In rats Medetomidine has also been reported to exert an initial increased in blood pressure followed by normalization [40]. We also observed that blood pressure was reduced after anesthesia and thereafter remained stable; that was the reason to obtain blood pressure measurements after 45 min. We do not have direct evidence of an increase in sympathetic activity in MUN rats, but it is likely that it occurs in this rat model, since it seems to be common to different animal models of fetal stress [18]. Therefore, under our experimental conditions, where sympathetic activity is blunted by anesthesia, it is possible that blood pressure in MUN rats, both in males and females might have been underestimated. MUN females remained normotensive at 22 months of age. However, there are some reports showing hypertension development at late stages of life in females exposed to other types of fetal stress [19,42]. Since hypertension development in females exposed to fetal stress factors seems to be related to sympathetic hyperactivation [43], we cannot rule out that, under our experimental conditions (in the presence of a reduced sympathetic activity by Medetomidine anesthesia) we failed to detect a modest increase in blood pressure in MUN females.

6-month old MUN males did not show signs of LVH measured by HW/BW. This was unexpected, given the fact of the initial hypertrophy found at weaning and the presence of high blood pressure, which is a well known stimulus for LVH [6]. Furthermore, at the age of 22 months MUN males exhibited increased HW/BW and echocardiographic signs of increased LV mass. Similar results have been described in male rats exposed to fetal low oxygen, i.e. an early heart hypertrophy, followed by normalization of HW/BW in the adult age and LVH at the age of 12 months [44]. These data support the hypothesis that different fetal insults lead to the development of similar cardiac alterations [18].

Oxidative stress is a possible mechanism responsible for the cardiac alterations found in the present study, although the enzymatic systems responsible for this disequilibrium between ROS production and elimination might be different in males and females. At the age of 6 months we found alterations in oxidative balance in MUN males due to an increase in XO, without a concomitant elevation of antioxidant enzymes. Female rats also showed oxidative disbalance, but in this case due to a generalized decrease in the enzymatic systems responsible for ROS degradation. In both cases an excess ROS is likely to take place and might be responsible for the hypertrophic response observed in ageing. A link between altered redox balance and cardiac hypertrophy has previously been reported in adult female offspring exposed to Na^+^ deficient diet during fetal life [45]. We cannot explain why MUN females exhibited a loss of antioxidant systems along maturation. We can suggest that the enzymatic systems responsible for ROS degradation might have been down-regulated due to a low ROS synthesis at early age (suggested by the observed reduction in NADPH oxidase expression), in a similar fashion as an increase in ROS production up-regulates antioxidant systems [46].

In both sexes, fetal undernutrition led to ventricular hypertrophy in old age, but the type of cardiac remodeling process seems to be different in MUN males and females. Thus, chamber dilatation was only observed in MUN females, but not in males, suggesting an eccentric growth. Hypertrophy was accompanied by reduction in ventricular function in both sexes, as shown by the lower ejection fraction in MUN rats. This functional loss might be related to excess collagen deposition which would reduce heart mechanical performance. It is possible that MUN heart exhibits increased collagen, since ROS elevation is known to participate in fibrosis and we found a pro-oxidative state in MUN cardiac tissue. Furthermore, increased fibrillar collagen has been reported in adult male rats exposed to fetal hypoxia and also in rats exposed to undernutrition [8], suggesting that it might be a common alteration induced by fetal stress. Cardiac fibrosis is likely to be larger in MUN males, due to the presence of both a pro-oxidative state from early age and hypertension, which is another stimulus for collagen synthesis. Hypertension might also be responsible for the observed increase in plasma BNP levels in aged MUN males, since natriuretic peptides, including atrial natriuretic peptide (ANP) and BNP, are released from the heart in response to overload [47]. Natriuretic peptides are considered biomarkers of cardiac disease, particularly in systolic heart failure. For example, circulating levels of these peptides correlate with echocardiographic evidence and with plasma markers of cardiac dysfunction in a pig model of malnutrition in early life [48]. Despite the similar reduction in ejection fraction in aged MUN males and females, plasma BNP was only elevated in males. These data suggest that males exposed to fetal undernutrition develop a larger degree of cardiac damage along ageing.

Regarding our initial hypothesis we conclude that females exposed to fetal undernutrition are better adapted than males at early stages of postnatal development and do not present cardiac alterations. However, in ageing females, like males, develop LVH and loss of heart function, although the severity of the cardiac damage might be lower, due to the absence of hypertension. These data, obtained in an experimental animal model, might explain the similar risk of heart disease in women exposed to fetal undernutrition, despite their relative protection against hypertension development.

## Supporting information

S1 Fig Representative gels of the different proteins tested. SOD, superoxide dismutases; Cu/Zn-SOD, Mn-SOD and extracellular SOD (EC-SOD); catalase; p22^phox^ and XO, xanthine oxidase, in cardiac tissue from 21-day and 6-month old male and female offspring from rats exposed to maternal undernutrition during pregnancy (MUN) and rats fed *ad libitum* (Control), relativized to GADPH expression.
(DOCX)

S2 Fig Representative blood pressure wave charts in anesthetized MUN and control rats at different age points. Blood pressure was recorded under Medetomidine/Ketamine anesthesia. MUN, rats exposed to maternal undernutrition during pregnancy; Control, rats fed *ad libitum*; M, males; F, females. Each chart represents 10 min recording period of the pressure wave at the beginning of the experiment (10 min after anesthesia) and at 45 min after anesthesia.
(TIF)
