# Supplementary figures and images for: Long term effects of fetal undernutrition on rat heart. Role of hypertension and oxidative stress
Source: PLoS One. 2017 Feb 17;12(2):e0171544. doi: 10.1371/journal.pone.0171544 (PMC5315302; doi:10.1371/journal.pone.0171544)

Supplemental material. Fig1





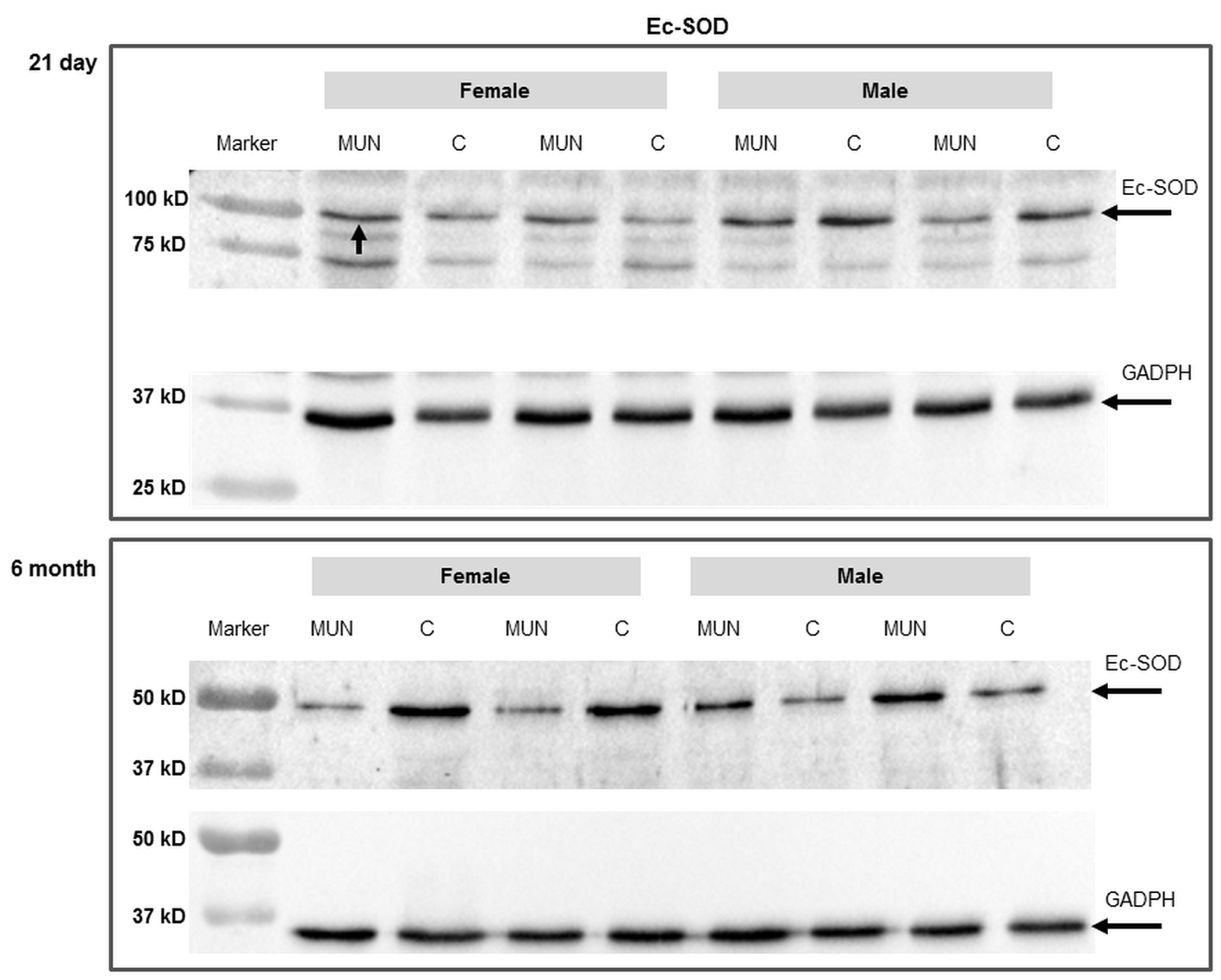


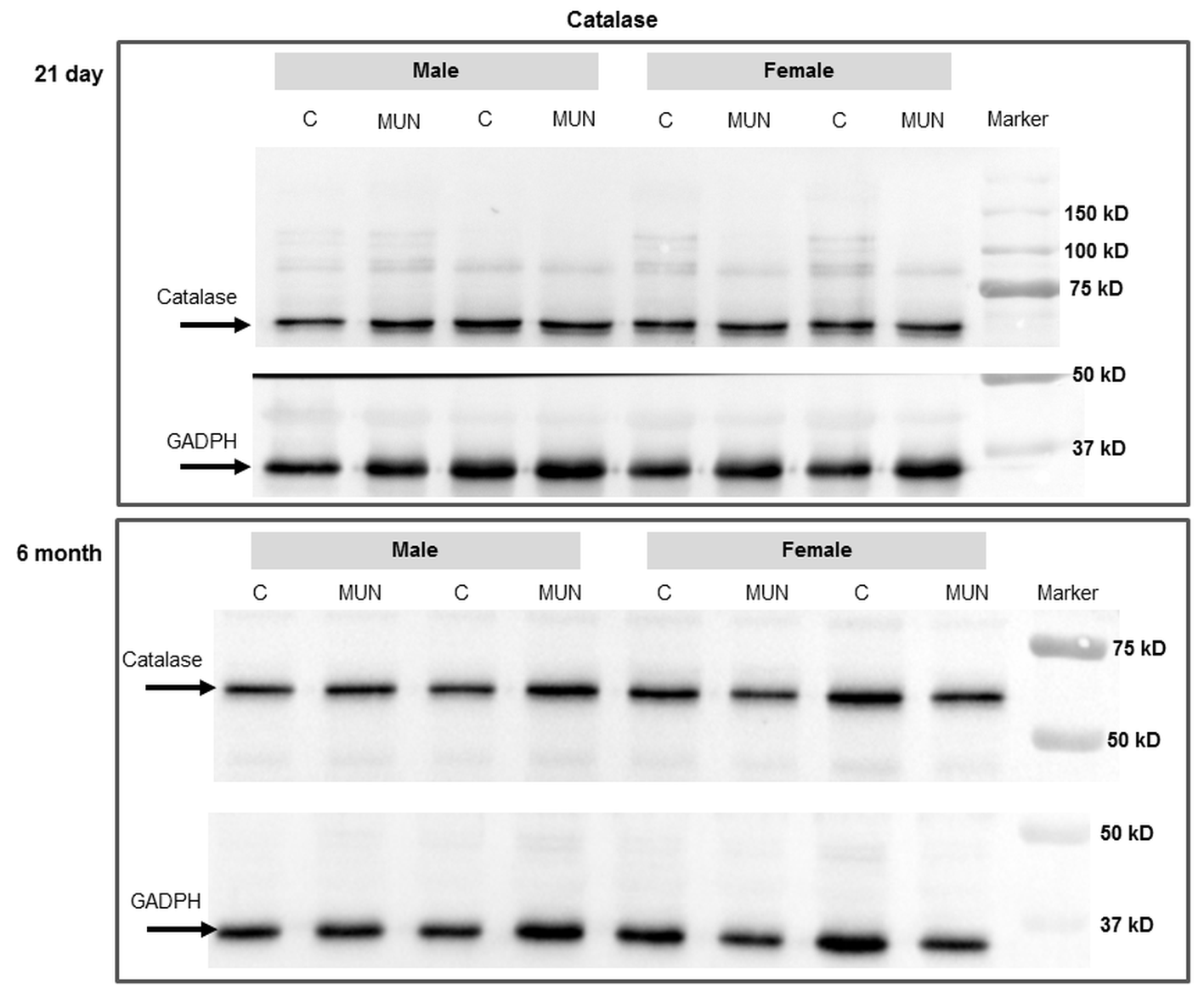


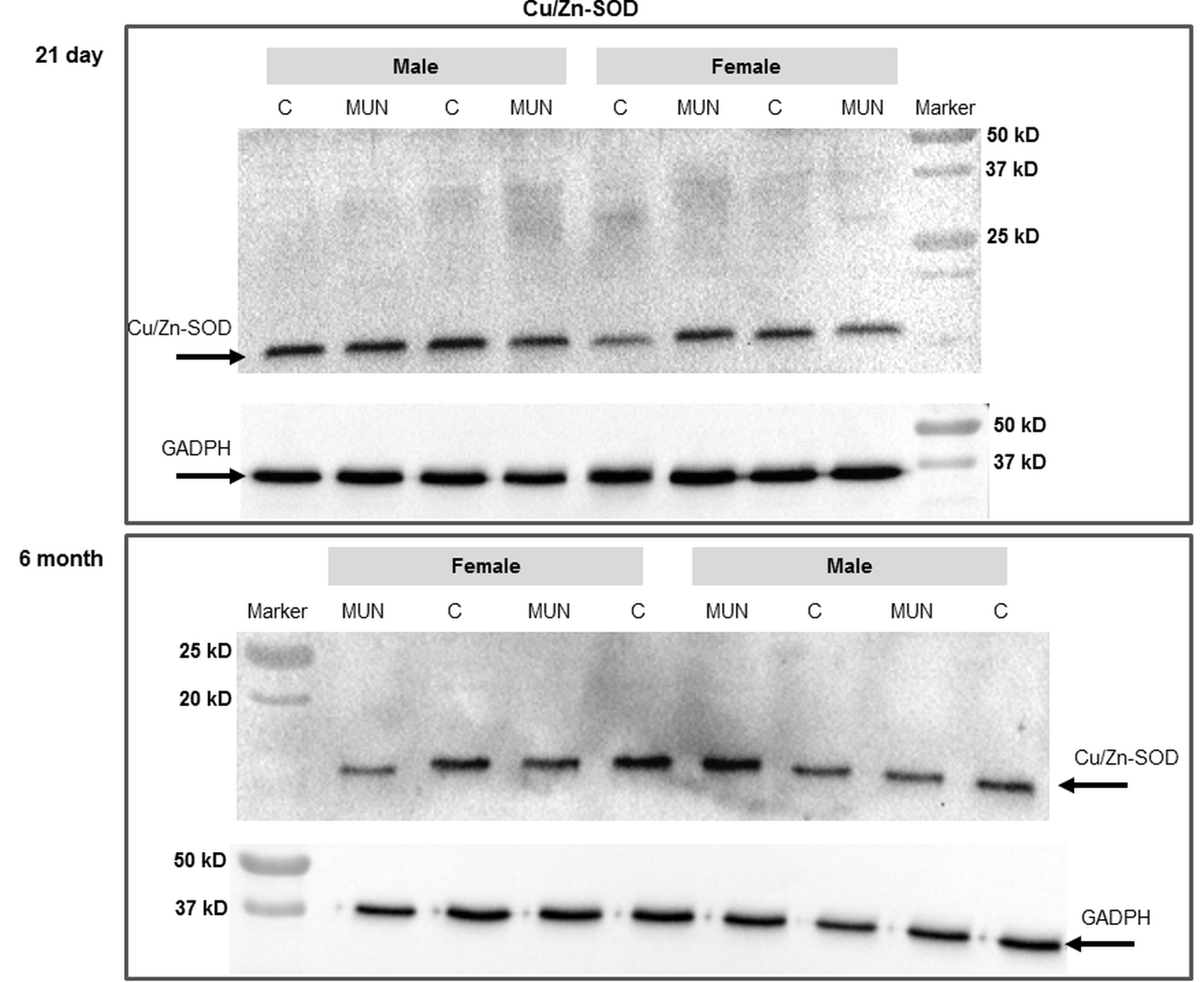


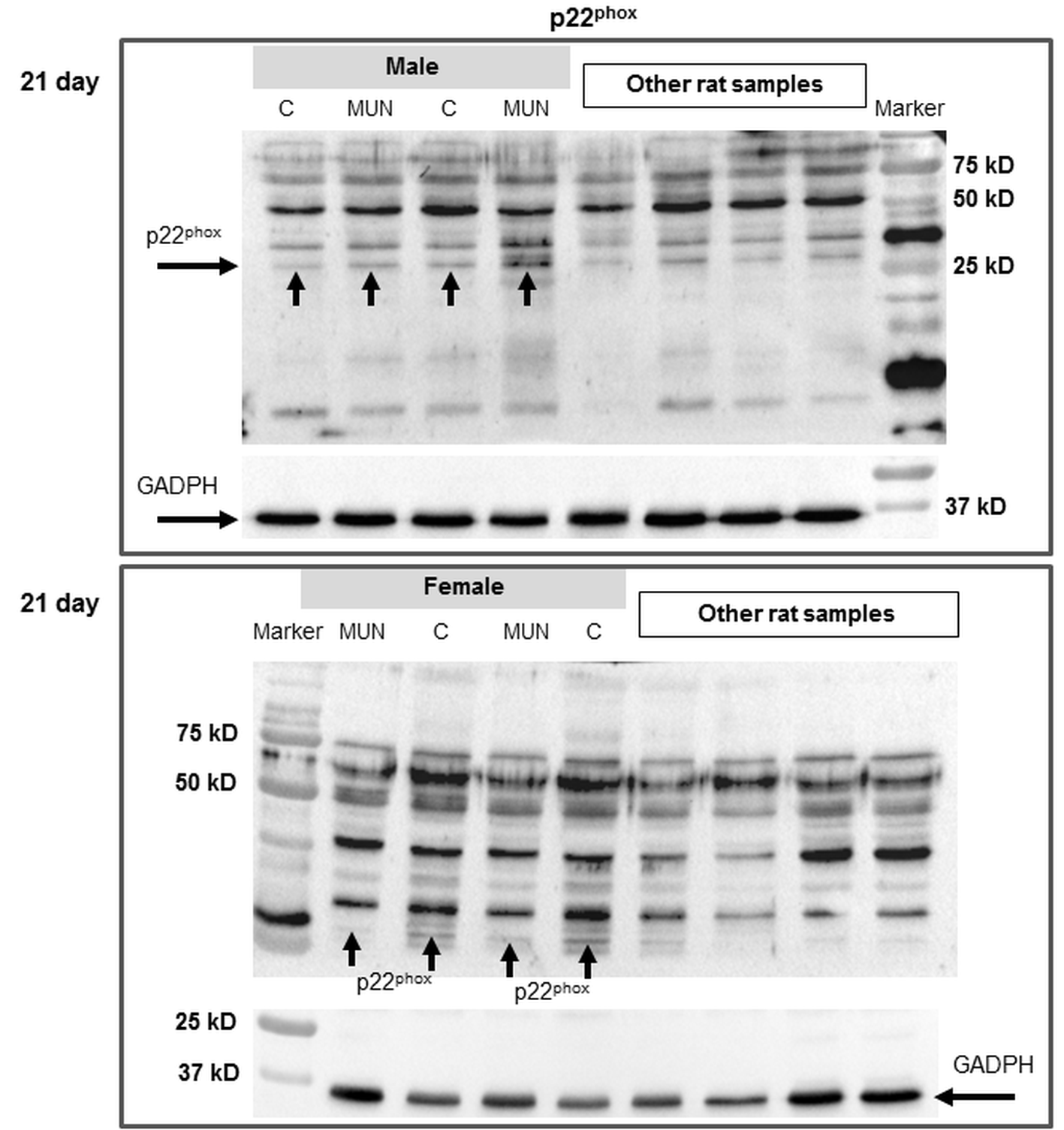


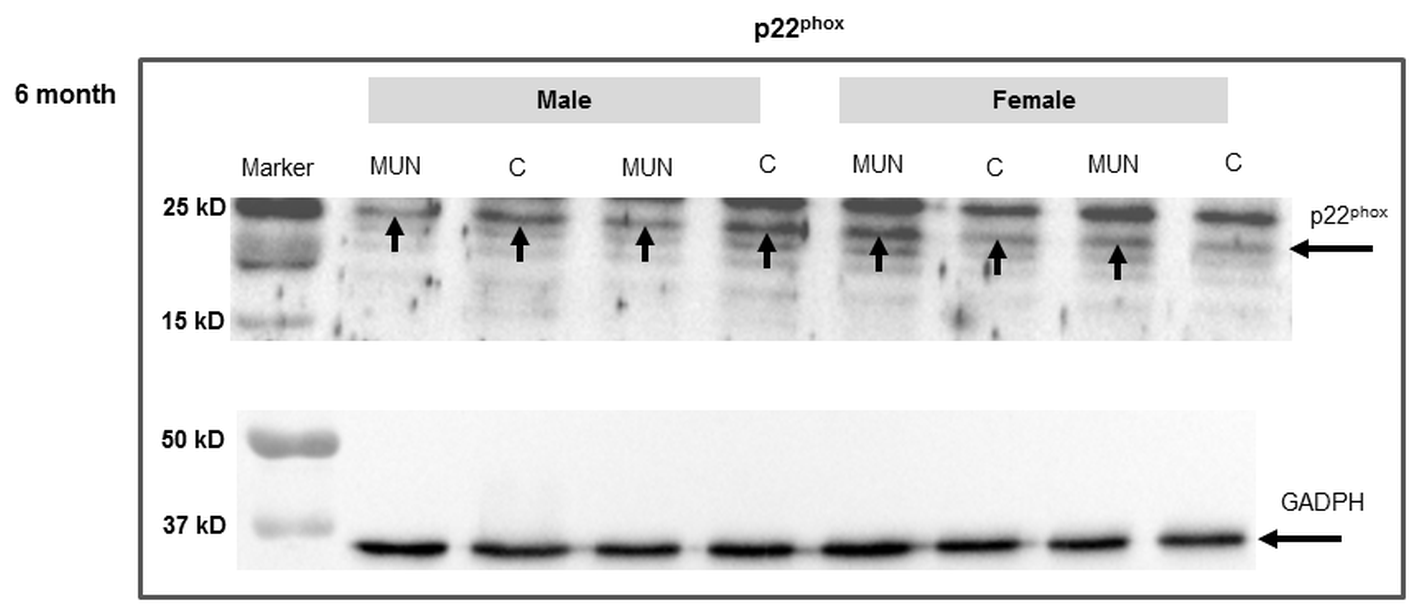


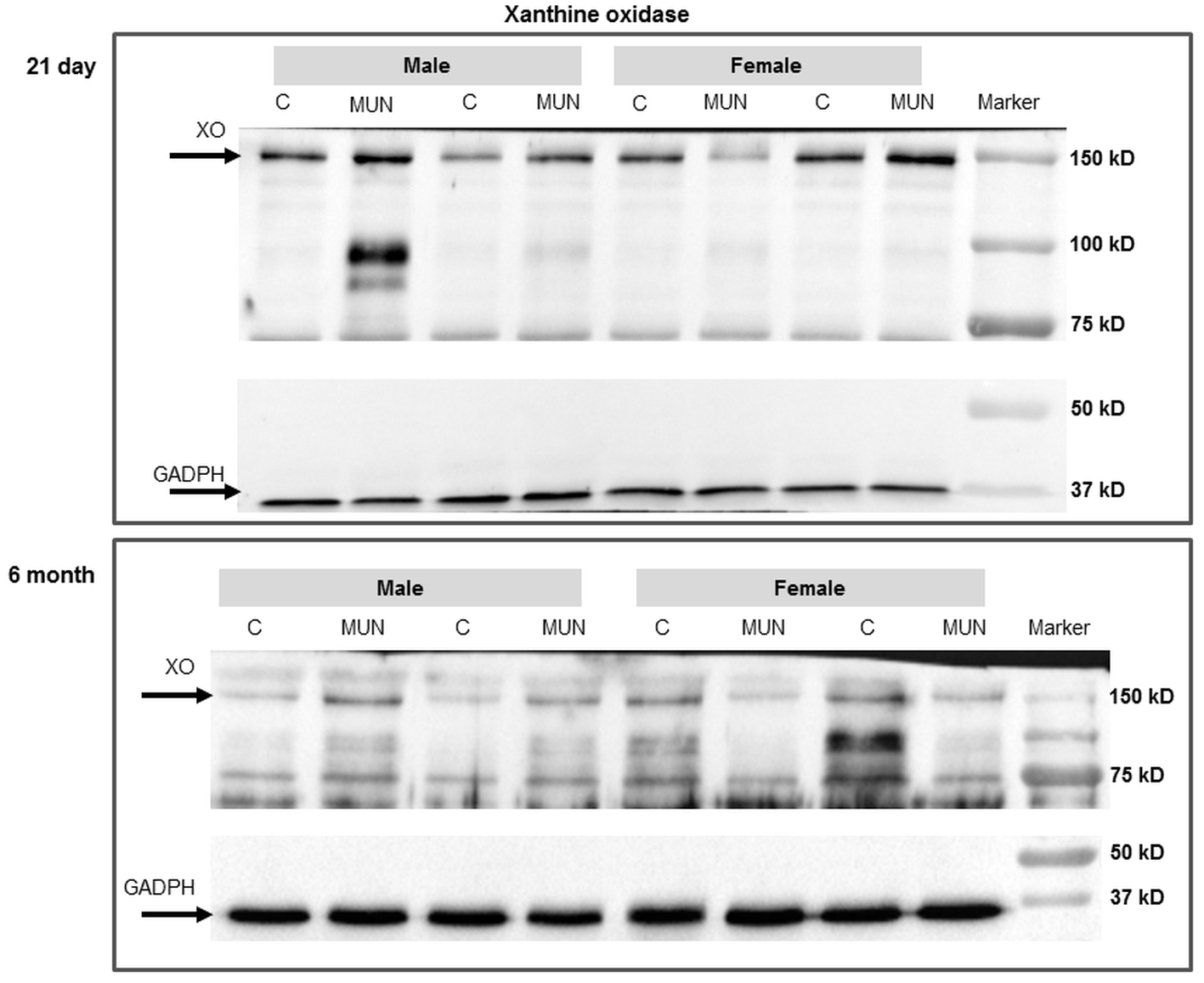

Supplement: S1 Fig — SOD, superoxide dismutases; Cu/Zn-SOD, Mn-SOD and extracellular SOD (EC-SOD); catalase; p22phox and XO, xanthine oxidase, in cardiac tissue from 21-day and 6-month old male and female offspring from rats exposed to maternal undernutrition during pregnancy (MUN) and rats fed ad libitum (Control), relativized to GADPH expression. (DOCX) [file pone.0171544.s001.docx]

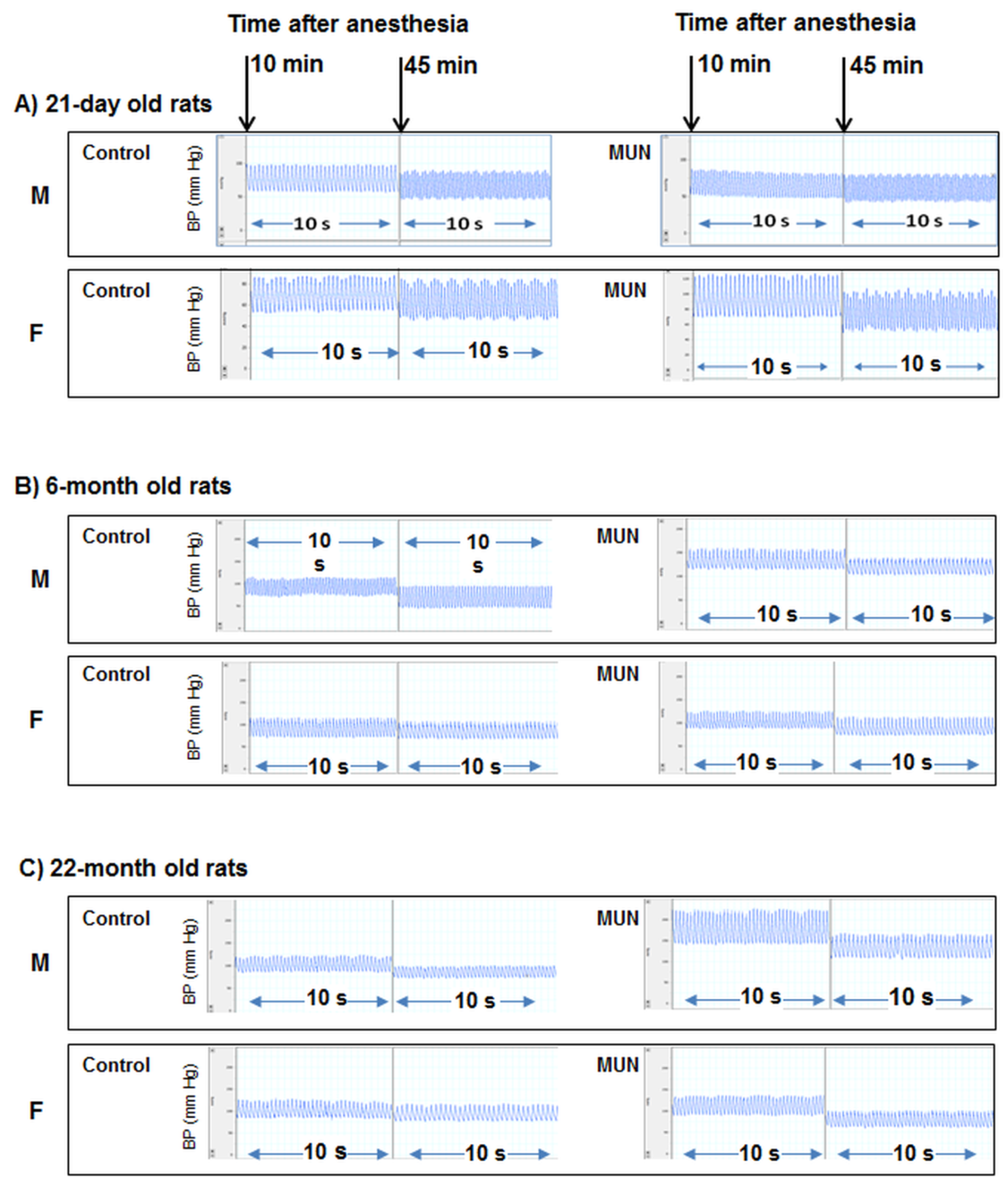

Supplement: S2 Fig — Blood pressure was recorded under Medetomidine/Ketamine anesthesia. MUN, rats exposed to maternal undernutrition during pregnancy; Control, rats fed ad libitum; M, males; F, females. Each chart represents 10 min recording period of the pressure wave at the beginning of the experiment (10 min after anesthesia) and at 45 min after anesthesia. (TIF) [file pone.0171544.s002.tif]
